# A Rare Case of Severe Neutropenia Due to Chikungunya Fever Which Improved With Filgrastim

**DOI:** 10.7759/cureus.20783

**Published:** 2021-12-28

**Authors:** Muhammad Rezeul Huq, Khaza Amirul Islam, Md. Aminur Rahman, Ahad Mahmud Khan

**Affiliations:** 1 Department of Neurology, Combined Military Hospital, Dhaka, BGD; 2 Department of Hematology, Shaheed Ziaur Rahman Medical College Hospital, Bogra, BGD; 3 Department of Hematology, Bangabandhu Sheikh Mujib Medical University, Dhaka, BGD; 4 Epidemiology, Projahnmo Research Foundation, Dhaka, BGD; 5 Usher Institute, The University of Edinburgh, Edinburgh, GBR

**Keywords:** granulocyte colony-stimulating factor, filgrastim, chikungunya fever, thrombocytopenia, severe neutropenia

## Abstract

Chikungunya fever is a re-emerging viral illness affecting different parts of the world. Most patients recover without any serious complications. Here, we present a rare case of chikungunya fever with severe neutropenia and moderate thrombocytopenia. A 31-year-old male presented with a fever, body aches, and rash. Serial full blood counts revealed a very low neutrophil count (0.273 × 10^9^/L) with a low platelet count (56 × 10^9^/L). Dengue fever was excluded by doing both antigen and antibody tests. The IgM antibody against the chikungunya virus was positive. After giving one dose of granulocyte colony-stimulating factor (G-CSF) filgrastim, the neutropenia resolved. A few days later, the thrombocytopenia resolved as well. Other than episodic attacks of arthritis, he recovered completely. The absence of severe neutropenia and thrombocytopenia is considered a major demarcating feature between chikungunya fever and dengue fever. Cases like this one put physicians in a difficult position regarding accurate diagnosis and appropriate management plans. The use of filgrastim may be considered as rescue therapy in a situation like this.

## Introduction

Both chikungunya fever and dengue fever are vector-borne diseases affecting mostly Africa and Asia for decades [1]. Recently, outbreaks of these viruses have shown unprecedented levels in Bangladesh, mostly in Dhaka city [2, 3]. Both diseases share some overlapping clinical and laboratory features. Also, outbreaks occur in the same season, making clinical differentiation difficult in some instances. However, the presence of marked myalgia, arthralgia, and the absence of cough, abdominal symptoms, or cytopenias go more in favor of chikungunya fever than dengue fever [4].

Neutropenia is classified by absolute neutrophil count as follows: mild neutropenia is defined as 0.5-1.5109/L, moderate neutropenia as 0.5-1109/L, and severe neutropenia as 0.5109/L [5]. Thrombocytopenia is classified as mild with a platelet count of 100-149 × 109/L, as moderate at 50-99 × 109/L, and severe with less than 50 × 109/L [6]. Though dengue fever may present with severe cytopenias and blood dyscrasias, we have not found such reported cases of chikungunya fever presenting with these features, making this case a rare one [7-9].

## Case presentation

**Figure 1:**
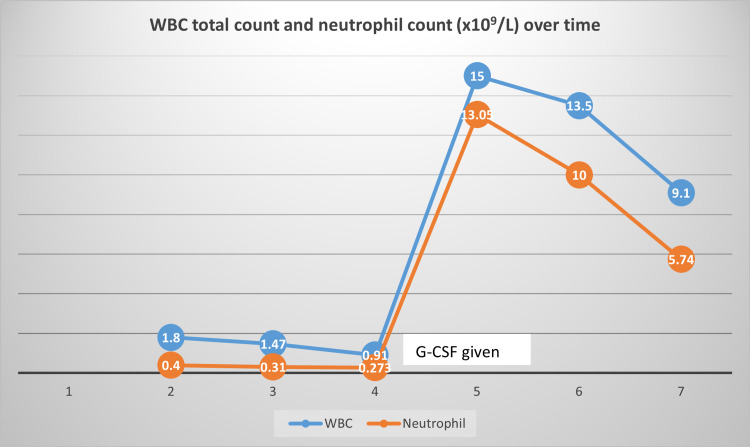
WBC total count and neutrophil count over time (days)

**Figure 2:**
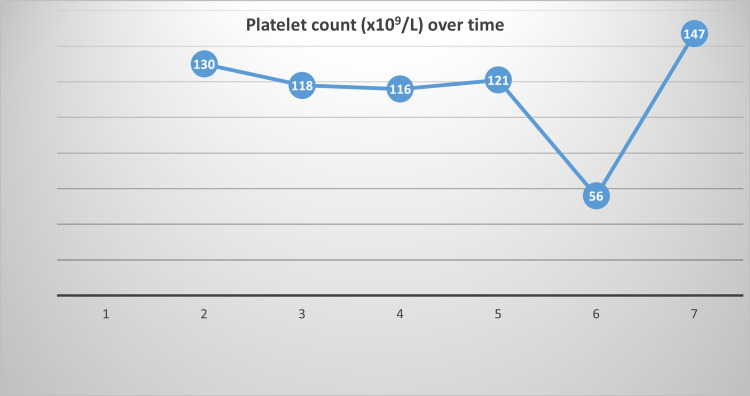
Platelet count over time (days)

A 31 years old male presented in 2017 with fever, body ache and rash for two days. Fever was high grade, intermittent with the highest recorded temperature 104-degree Fahrenheit. It was preceded by some prodromal features like malaise, myalgia for one day. The patient also suffered from a generalized erythematous macular rash all over the body, which was itchy and blanched with pressure. Generalized weakness and body ache were so severe that the patient became almost bedbound. He consulted in the outpatient department (OPD) of Bangabandhu Sheikh Mujib Medical University (BSMMU), and investigation revealed leucopenia (1.8 × 10^9^/L) with severe neutropenia (0.4 × 10^9^/L) and mild thrombocytopenia (130 × 10^9^/L) (day two after the onset of fever). Dengue NS1 antigen test was negative. The next day, the severity of leucopenia (1.47 × 10^9^/L) and neutropenia (0.31 × 10^9^/L) increased. He was admitted for further monitoring and management. Fever was still present as before, and he developed some features of immunosuppression like herpes zoster ophthalmicus and oral thrush. Empirical broad-spectrum antibiotics, oral antiviral and topical antifungal were started. Other than fever, his vitals were stable. On the next day, the total count of white blood cells (WBC; 0.91 × 10^9^/L) and neutrophil count (0.273 × 10^9^/L) went further downward. The serum ferritin level was very high (6521.0 ng/ml). Blood culture, urine routine examinations and culture revealed no abnormalities. Also, serum enzyme levels- serum glutamic pyruvic transaminase (SGPT; 260 U/L), serum glutamic-oxaloacetic transaminase (SGOT; 204 U/L) and lactate dehydrogenase (LDH; 461 U/L) were raised. The patient was given one dose of filgrastim (30 MIU), and next day neutrophilic leucocytosis (15 × 10^9^/L total count with 87% neutrophil) was found. On subsequent days, though, WBC and neutrophil count normalized, platelet count went further downward (56 × 10^9^/L). But, the patient improved clinically by this time. Chest imaging and abdominal sonography were also normal. Eventually, all the blood parameters became normal (Figures 1, 2). After seven days of onset of fever, the anti-dengue IgM antibody was done, and it was negative. Chikungunya IgM antibody test by rapid immunochromatographic test (ICT) was a positive indicating acute infection. His other family members were also symptomatic and positive for the chikungunya serology test. Blood enzymes and serum ferritin levels were also within normal limits after a few weeks. He was discharged after five days of hospitalization. The patient recovered completely except periodic attacks of flitting inflammatory arthritis involving his wrist and ankle joints with plantar fascitis over the next one year.

## Discussion

Chikungunya fever is confirmed if the patient meets one or more of the following findings, irrespective of the clinical presentation as proposed by the World Health Organization (WHO) Regional Office for Southeast Asia: virus isolation in cell culture or animal inoculations from acute-phase sera; presence of viral ribonucleic acid (RNA) in acute-phase sera as determined with RT-PCR; presence of virus-specific IgM antibodies in single serum samples in the acute phase; four-fold increase in virus-specific IgG antibody titer in samples collected at least three weeks apart [10].

IgM antibodies can be detected by an ICT or an enzyme-linked immunosorbent assay (ELISA). Both the tests have low sensitivity but high specificity in detecting acute infections [11]. In our case, we confirmed the diagnosis by detecting IgM antibodies by the ICT method. Due to less availability, virus isolation or RT-PCR could not be done. 

Viral infections are among the most common causes of neutropenia, due either to bone marrow suppression or peripheral destruction. The agents commonly implicated include Epstein-Barr virus, cytomegalovirus, hepatitis A and B viruses, parvovirus, influenza virus species, and measles [12].

Chikungunya fever is not a common cause of neutropenia. In one study, neutropenia and thrombocytopenia were regarded as negative predictors of chikungunya fever [4]. In another study, none of the chikungunya fever cases was found to have a neutrophil count of less than 1 × 109/L [13].

Most of the guidelines recommend hospitalization for severe neutropenic patients with empirical antimicrobials therapy. The use of granulocyte colony-stimulating factors (G-CSFs; also referred to as hematopoietic growth factors) like filgrastim is also well known, especially in chemotherapy-induced neutropenia [14]. As universal management protocols for viral infection-induced severe neutropenia are still lacking, clinicians often follow similar protocols for chemotherapy-induced neutropenia. There are a few other case reports of the usage of G-CSF as rescue therapy in severe neutropenia due to viral infection [15]. In this case, the patient developed some features of immunosuppression requiring antimicrobial therapy. And the increasing severity of neutropenia led us to the usage of filgrastim as rescue therapy. The dosage and duration of filgrastim depend upon the indications, and as there is no established regimen regarding its use in viral infection, we used a single standard dose, and the neutrophil count improved the next day. Thrombocytopenia did not need any specific management and recovered spontaneously. 

Interestingly, our patient had a very high level of serum ferritin initially, which later came to a normal level. In one study, in chikungunya fever, a significant increase in serum ferritin level was found in viraemic patients compared to non-viraemic patients. Besides, the proportion of patients with prolonged chronic arthralgia (≥3 months) was significantly higher in the group with high ferritin levels compared to normal ferritin levels. Our patient also suffered from chronic arthralgia and arthritis for about one year [16].

## Conclusions

We need more data to include chikungunya fever among the causative agents of severe neutropenia. Co-infection with dengue fever should be excluded, as dengue is a known etiological agent for severe cytopenia. Formulation of a management protocol for viral infection-induced severe neutropenia is a crying need. Randomized controlled trials may dictate the use of filgrastim in severe neutropenia due to viral illness in the future.
